# Interconnected Adaptive Responses: A Way Out for Cancer Cells to Avoid Cellular Demise

**DOI:** 10.3390/cancers14112780

**Published:** 2022-06-03

**Authors:** Gabriella D’Orazi, Mara Cirone

**Affiliations:** 1 Department of Neurosciences, Imaging and Clinical Sciences, University “G. D’Annunzio”, 66013 Chieti, Italy; gadorazi@unich.it; 2 Unit of Cellular Networks, Department of Research and Advanced Technologies, IRCCS Regina Elena National Cancer Institute, 00144 Rome, Italy; 3 Department of Experimental Medicine, University of Rome LA Sapienza, Viale Regina Elena 324, 00161 Rome, Italy

**Keywords:** ER stress, UPR, DDR, HSPs, autophagy, anti-oxidant response, cancer, death resistance

## Abstract

**Simple Summary:**

One of the major obstacles to anti-cancer therapy is the development of drug resistance that allows the cancer cell to adapt to the treatments and keep surviving. In this mini-review we attempt to give an overview of the adaptive responses and the cross-talk between them that could be targeted to counteract cancer resistance to stress and improve the outcome of cancer treatment.

**Abstract:**

Different from normal cells, cancer cells must hyperactivate a variety of integrated responses in order to survive their basal stress or its exacerbation caused by exposure to anti-cancer agents. As cancer cells become particularly dependent on these adaptive responses, namely UPR, DDR autophagy, anti-oxidant and heat shock responses, this turns out to be an Achille’s heel, which allows them to be selectively killed while sparing normal unstressed cells. Better knowledge of the cross-talk between these adaptive processes and their impact on the immune system is needed to design more effective anti-cancer therapies, as reviewed in this paper.

## 1. Introduction

All living cells, by their nature, attempt to defend themselves from attacks coming from the intracellular or extracellular environment in order to preserve cellular homeostasis. However, this is possible until the damage becomes so severe that cellular demise becomes an unavoidable choice [1]. These few words recapitulate a variety of extremely intricate processes and molecular pathways whose interplay dictates the final cell fate. To reach the ideal goal of killing cancer cells and concomitantly sparing normal, non-transformed cells, it is of fundamental importance to understand the differences between them and address the appropriate target treatments. One of the most relevant differences between cancer and normal cells is the condition of constitutive stress. The formers are forced to live due to their intense cell proliferation, the presence of oncogenes, the shortage of nutrients and the exposure to an inflammatory and/or hypoxic environment. To adapt to stress, cancer cells must hyperactivate a variety of responses from which they become strongly dependent. Therefore, targeting them could represent a promising avenue in anti-cancer therapy. Among these adaptive responses, there is the Unfolded Protein Response (UPR) [2], triggered in response to endoplasmic reticulum (ER) stress and initiated by three sensors, namely Inositol-requiring enzyme 1 (IRE1)alpha, Activating Transcription Factor 6 (ATF6) or protein kinase RNA-like endoplasmic reticulum kinase (PERK) [3], and the DNA Damage Response (DDR) that encompasses several pathways, activated depending on types of DNA damage and on the repair machinery available in the particular moment in which DNA damage occurs [4]. UPR and DDR are not only strictly interconnected with each other [5]. Still, they communicate with other adaptive processes, also activated in response to stress, namely autophagy, the anti-oxidant and heat shock response [6,7]. This mini-review will discuss how targeting UPR, DDR, the interconnection between them and the other above-mentioned adaptive processes could offer the opportunity to selectively kill cancer cells.

## 2. Stress Response Pathways and Cancer Survival

### 2.1. UPR and Cancer

**Figure 1:**
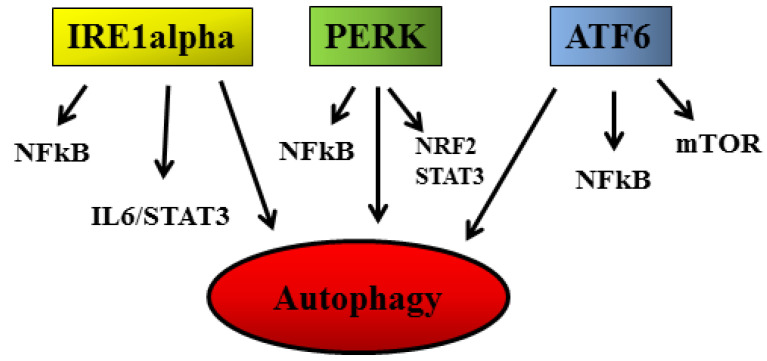
Role of UPR sensors (IRE1alpha, PERK; and ATF6) in the induction of autophagy and in the activation of NFkB, IL6/STAT3, NRF2 and mTOR pro-survival pathways.

Given the constitutive condition of stress that characterizes cancer cells, their exposure to anti-cancer treatments that induce organelle or macromolecule damage exacerbates such stress and renders it no longer bearable for the cell. Interestingly, the same treatments can induce milder stress in normal, non-transformed cells that are still able to activate protective processes. The different intensity of ER stress may, for example, result in the activation of opposite functions of UPR [8], skewing the UPR balance towards cell death in cancer cells [9] while activating the adaptive functions of UPR in normal cells. Therefore, this plasticity of UPR can be exploited to selectively kill cancer cells, in which the pro-apoptotic molecule C/EBP Homologous Protein (CHOP) may result up-regulated, mainly through PERK signaling. Among its pro-apoptotic functions, CHOP leads to an unbalance between the anti-apoptotic and pro-apoptotic Bcl-2 family proteins [3] and increases the expression of the death receptors (DR) 5, to promote apoptosis [10]. However, the use of anti-cancer treatments that kill cancer cells by exacerbating ER stress requires special caution, as the intensity of stress can influence the exposure or the release of molecules such as damage-associated molecular patterns (DAMPS), that strongly affect the anti-cancer immune response, essential for tumor eradication [11,12,13]. Interestingly, it is possible to exacerbate ER stress and skew UPR balance toward cell death by selectively inhibiting one or more UPR sensors in cancer cells [8]. This is possible because, as mentioned above, due to the constitutive stress, these cells strongly rely on UPR to maintain their survival, particularly on the activation of ATF6 and IRE1 alpha/XBP1s arms [14]. Among other the numerous functions, the signaling initiated by these two sensors increases the expression of ER chaperones such as 78-kDa glucose-regulated protein (Grp78)/BiP, which play a key role in protein refolding and promote mRNA degradation as well as misfolded protein elimination via ER-associated degradation (ERAD) or autophagy [15,16]. The latter process contributes to PERK signaling, which also has other pro-survival functions, such as blocking protein translation and activating the anti-oxidant response through nuclear-factor-erythroid 2-related (NRF2) phosphorylation (Figure 1) [17]. It is important to consider that the signaling initiated by the three UPR sensors (e.g., IRE1 alpha, PERK and ATF6) may result in the activation of pro-survival pathways such as nuclear factor kappa B (NFkB), interleukin 6 (IL6)/STAT3 (signal transducer and activator of transcription 3), NRF2 and mammalian target of rapamycin (mTOR) [18]. Interestingly, these pathways can, in turn, influence ER stress and UPR activation in a tangle of intricate regulatory circuits also involving autophagy [19,20,21] (Figure 1).

Of note, STAT3 [22] and mTOR [23] can strongly regulate autophagy, a catabolic process articulated in different steps that, to some extent, also helps to preserve cancer cell integrity and to resist nutrient shortage [24]. As mentioned for UPR, autophagy is usually basally hyper-activated in cancer cells in comparison to normal cells [25]. It is further stimulated in response to several cytotoxic treatments, continuing to play a pro-survival role. Indeed, autophagy inhibition can represent a strategy to improve the outcome of anti-cancer therapy [26]. However, it must be considered that the role of autophagy can vary depending on the different phases of carcinogenesis [24]. Different from what occurs in established cancers, autophagy may prevent cancer onset as, through this catabolic process or the selective forms of it, toxic materials and damaged organelles such mitochondria, producing reactive oxygen species (ROS) and causing DNA damage, can be eliminated [27].

**Figure 2:**
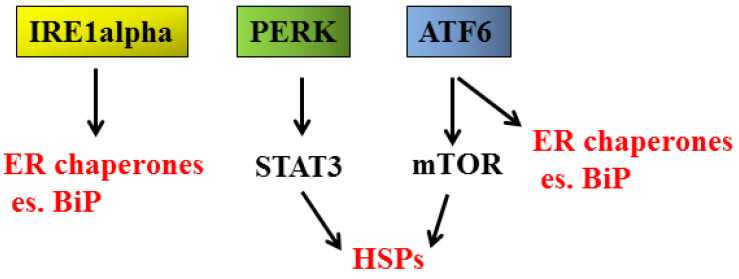
Role of UPR sensors (IRE1alpha, PERK; and ATF6) in the up-regulation of heat shock proteins (HSPs).

Next, we come to another cellular response, the anti-oxidant response, which is reported to play different roles, depending on the phase of carcinogenesis, preventing cancer initiation while sustaining the survival of established cancers [28]. The most important molecule in charge of mediating the anti-oxidant response is NRF2, classically activated by oxidative stress or, as mentioned above, following phosphorylation by the PERK sensor (Figure 1) [29]. NRF2 can also be stabilized by p62/SQSTM1 [30], protein accumulating as a consequence of autophagy inhibition. Through the activation of NRF2, UPR may help to prevent cancer formation. Still, on the other hand, it must be considered that UPR activation promotes the release of pro-inflammatory cytokines as well as inflammatory/immunosuppressive molecules such as prostaglandin E2 (PGE2), promoting carcinogenesis [13,31,32,33]. Indeed, a strict inter-connection between long-lasting/chronic inflammation and cancer onset exists, in which STAT3, NFkB and mTOR, pathways regulating both inflammation and cell proliferation, are key players [34]. Finally, UPR cross-talks with the heat shock response (HSR) [35] (Figure 2), orchestrated by heat shock proteins (HSPs), a family of proteins classified based on their molecular weight, from small HSPs such as HSP27 to high molecular weight HSPs such as HSP90 [36]. Besides heat shock factor 1 (HSF1), which represents the main factor regulating their transcription, HSPs expression is regulated by pathways such as mTOR [37,38], both activated through UPR signaling. Regarding this topic, we have recently shown that STAT3, by up-regulating HSP90, promoted mutant p53 (mutp53) stabilization [39], unveiling another important mechanism through which STAT3 sustains carcinogenesis. Interestingly, the reverse is also true, as mutp53 has been reported to sustain STAT3 activation [40]. Of note, we have previously shown that the inhibition of STAT3 activated wild-type (wt) p53 in lymphoma cells undergoing apigenin treatment [41], adding this to the numerous examples in which the same treatment induces opposite effects on wt vs. mutp53.

### 2.2. DDR and Cancer

Another promising strategy to preferentially target cancer cells is to interfere with DNA repair, orchestrated by several pathways, from which cancer cells are strongly dependent [4]. Indeed, DNA breaks often occur spontaneously in cancer cells. Due to their high proliferation rate, they more frequently enter mitosis, a phase in which the DNA repairing systems are less active [42]. To the DNA damage response (DDR), belong molecules able to detect DNA damage, molecules able to block cell cycle and several others that perform the DNA repair or induce death [43]. The DNA repair pathways comprise the direct DNA damage reversal, base excision repair (BER), mismatch repair (MMR), nucleotide excision repair (NER), homologous recombination (HR) and non-homologous end-joining (NHEJ) that repair the DNA double-strand breaks (DSBs). Although these processes mainly take place in the nucleus, it must be considered that some DDR pathways, particularly BER, are also active in the mitochondria [44]. A variety of small molecules able to inhibit the molecules involved in DNA repair, from those repairing the single-strand break (DDBs) to those mediating the DNA double-strand break (DSBs) repair, have been discovered and tested against cancer [45]. The inhibitors of poly(ADP-ribose) polymerase (PARP) family proteins seem to be very promising, given that PARPs contribute to almost all DNA repair pathways, from BER and NER to the HR and NHEJ repair [46], the latter being active in all cell cycle phases, while the former is predominantly active in S/G2 phase of the cell cycle. The inhibitors of PARPs or those targeting other molecules involved in DDR have been shown to exert a stronger cytotoxic effect against cancer, particularly in combination with DNA damaging agents, such as radiations or genotoxic drugs [47,48], strategies that allow reducing the doses of these treatments and thus, the side effects that they often cause. The synergic cytotoxic effect may be due to the fact that such anti-cancer treatments induce DNA breaks and consequently increase the dependence of cancer cells on DDR for their survival. Regarding cancer onset, while the role of UPR sensor activation in the first carcinogenesis steps remains to be fully clarified, a proper function of DDR is unequivocally required to prevent oncogenic transformation. The clearest evidence that DDR activity is essential to prevent cancer onset is that cancers more frequently arise in patients carrying mutations of DDR molecules, from those regarding onco-suppressor p53, to those regarding ataxia telangiectasia mutated/ATM), ataxia telangiectasia and Rad3-related protein (ATR), BRCA-1 and so on [49].

**Figure 3:**
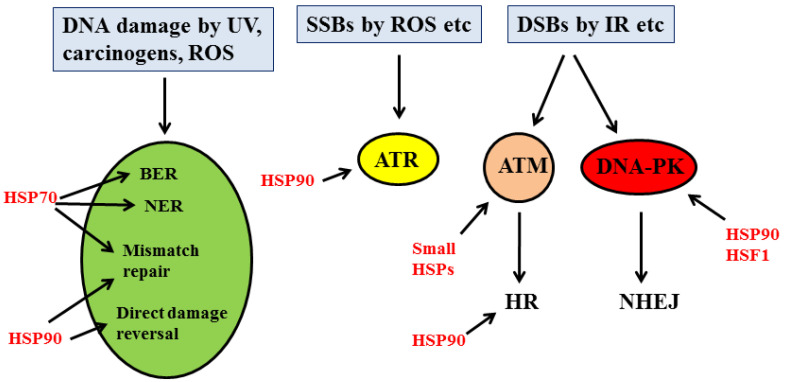
Interaction between DDR molecules and HSPs in response to DNA damage.

**Figure 4:**
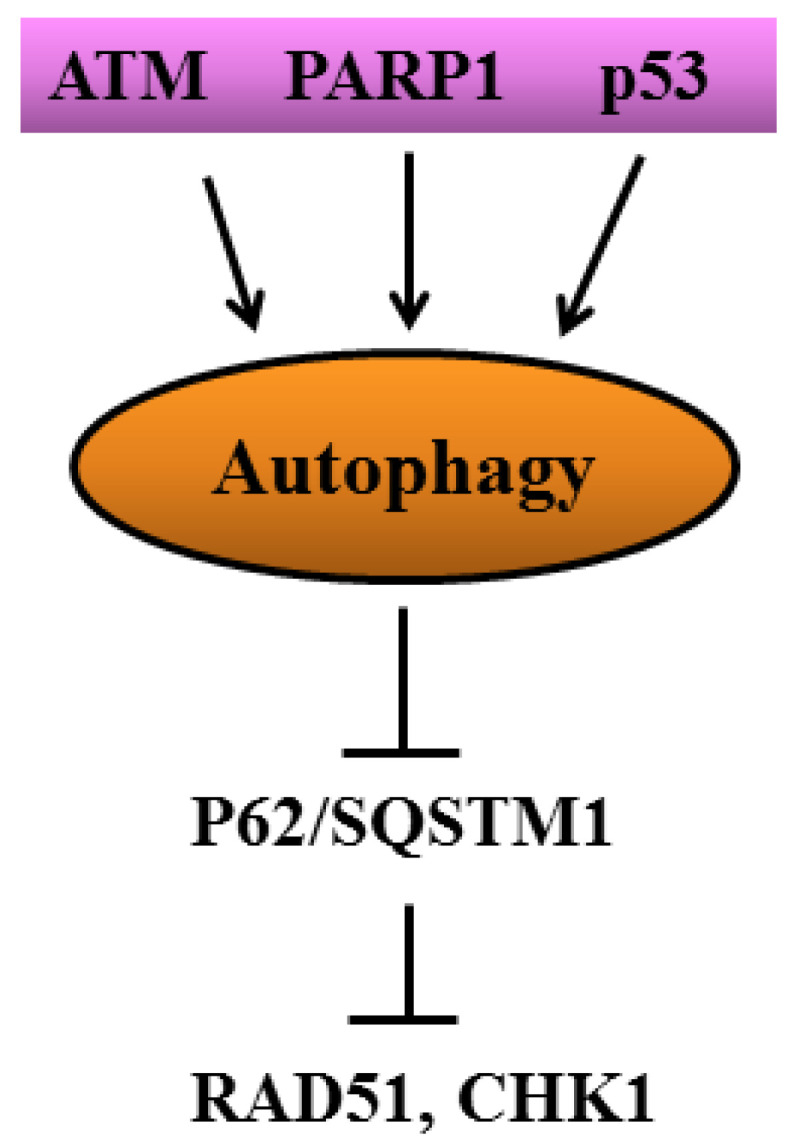
Cross-talk between DDR molecules and autophagy.

Another protective response strictly interconnected with DDR is the Heat Shock Response (HSR). Several members of HSPs have been shown to interact and promote the stability of many DDR proteins, considered to be HSP client proteins. The kinases ATM, ATR and DNA-dependent protein kinase (DNA-PK), as well as the molecules responsible for cell cycle arrest, such as CHK1, mainly interact with HSP90 subfamily proteins, while those involved in BER and NER mainly associate with HSP70. Proteins mediate the double-strand break repair, such as BRCA1 and RAD51, that rely on HSP90, while the Ku80/Ku70 complex belonging to the NHEJ pathway is associated with small HSPs [50] (Figure 3). This suggests that targeting HSPs could represent a strategy to interfere with DDR. However, caution should be taken when targeting HSPs, considering that their role in carcinogenesis seems to be controversial [51]. Of note, DDR is strictly linked to autophagy. The latter process has been reported to regulate DDR [52], and autophagy may be triggered by DDR activation at multiple levels. Indeed, ATM can induce autophagy by phosphorylating AMPK and inhibiting mTOR, and p53, which plays a key role in DDR, may trigger autophagy via Sestrin 1, Sestrin 2, DRAM or by up-regulating PTEN and de-phosphorylating mTOR [53] (Figure 4). Finally, DDR is interconnected with the autophagic process through p62/SQSTM1. This stress-inducible protein accumulates as a result of autophagy inhibition and is known to be involved in cancer progression by several means, including the interaction with NRF2, mTOR and NFkB [54,55]. Regarding DDR, p62/SQSTM1, when localized in the nucleus, has been shown to promote the proteasomal degradation of HR molecules such as RAD51, skewing of DNA repair balance towards NHEJ, an error-prone DNA repair mechanism [56] (Figure 4). However, in primary B lymphocytes undergoing EBV-driven transformation, we found that p62/SQSTM1 played a tumor-preventing role by sustaining the expression of DDR molecules such as ATM and H2AX stabilizing NRF2, reducing inflammation and promoting mitophagy [57].

### 2.3. UPR/DDR Interplay and Cancer

**Figure 5:**
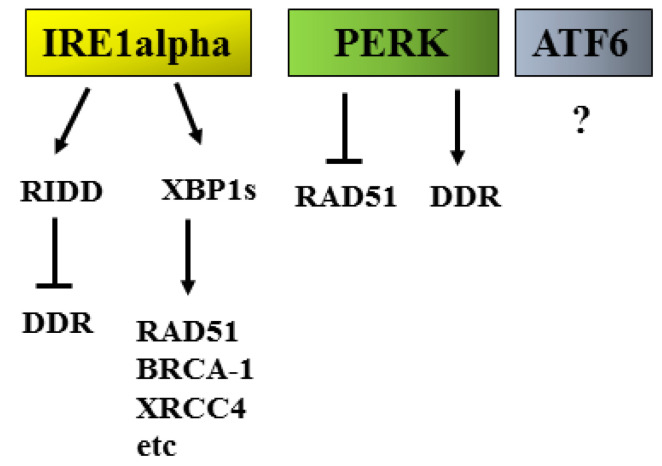
Cross-talk between UPR (IRE1alpha, PERK, ATF6) and DDR molecules.

Although UPR takes place in the ER while DDR mainly occurs in the nucleus, these two adaptive responses are strongly interconnected [5]. This implies that pathological changes occurring in the cytoplasm can be sensed by the nucleus and vice versa. Several studies have explored the interplay between ER stress/UPR and DDR (Figure 5), showing that the induction of ER stress and PERK activation can reduce the capacity of cells to repair DNA damage, inducing the degradation of RAD51 [58,59]. However, PERK has been reported to also sustain DNA repair in other cancer cell types, increasing radio-resistance [60].

It is emerging that the manipulation of UPR may affect the expression or activity of molecules belonging to DDR. It is particularly important to assess the impact of each UPR sensor on DDR molecules in order to design the most appropriate combination therapies able to counteract the mechanisms that allow cancer cells to survive, despite the exposure to anti-cancer treatments. Interestingly, some molecules belonging to UPR, besides ER stress, can be activated by DNA damage [61]. For example, ATF4, a transcription factor involved in the up-regulation of the pro-apoptotic molecule CHOP, can be activated by both UPR and DDR signaling. Interestingly, CHOP may, in turn, sustain the transcription of DUSP5 to mediate the dephosphorylation of ERK1/2 [62], a kinase able to preserve cancer cell survival either in the course of ER stress and/or genotoxic stress [63,64]. ERK1/2 may indeed counteract the pro-apoptotic function of wtp53 [59,65], another protein that bridges UPR to DDR. Of note, p53 contributes to the transcription of DUSP5 and thus to the inhibition of ERK1/2 [66] in a negative feedback loop. Among the UPR arms, the most strictly involved in the regulation of DDR molecules is IRE1alpha. Through its endoribonuclease activity on XBP1, it generates XBP1s, a transcription factor that controls the expression of HR proteins such as RAD51 and BRCA-1 but also those belonging to the NHEJ pathway such as XRCC4 [5]. Moreover, Ire1alpha can influence DDR also through its RIDD, as several mRNAs encoding for DDR proteins are regulated by this activity. The other way around may also occur, as genotoxic agents that induce DNA damage have been reported to affect IRE1 alpha activity, reducing the splicing of XBP1 and the regulated Ire1 dependent decay (RIDD) [67] or, in other cases, engage the RIDD activity of IRE1 alpha [68]. At this regard, we have recently found that the combination of PARP and CHK1 inhibitor reduced XBP1s that in turn downregulated BRCA-1, to perpetuate DNA damage, and promoted autophagy in myeloma and lymphoma cells [69]. It has been reported that quercetin [70] could potentiate the anti-cancer effect of DNA damaging agents. It will be interesting to investigate the impact of natural compounds known to induce ER stress on the expression/activity of DDR molecules. 

Regarding the interplay between UPR and DDR, the less investigated UPR sensor is ATF6, and we are currently focusing on its role in the activation/inhibition of DDR. Moreover, the role of ATF6 on bulk macroautophagy and in its particular forms, such as the chaperone-mediated autophagy (CMA), is under investigation in our laboratory. These processes may indirectly affect DDR by contributing to the degradation of mutant p53 [71,72]. Interestingly, mutp53 is able to sustain ATF6 activation to increase the resistance of cancer cells to stress [73]. CMA is a process that involves HSPc70, and this highlights once again the key role of HSPs in the intricate network of the adaptive processes that sustain cancer survival, even if CMA, when leading to the degradation of oncogenes, can prevent carcinogenesis rather than promoting it. In particular, as CMA contributes to the regulation of c-myc expression level, it not only influences DDR, sustaining the expression of RAD51 and BRCA-1 but also affects UPR, promoting its pro-survival functions [74,75]. Another important aspect to be considered is that besides UPR, DDR has been reported to influence the release of inflammatory cytokines [74,76], thus, contributing to shaping the tumor microenvironment. It will be interesting to explore whether the effects on the release of cytokines mediated by DDR manipulation could occur through its interplay with ER stress/UPR or independently of it by using molecules able to reduce ER stress such as 4-Phenylbutyric acid (4-PBA) or to inhibit the single UPR sensors. This aspect is particularly important when considering that the tumor microenvironment strongly influences the anti-cancer immune activity of macrophages, dendritic cells and lymphocytes and can modify these cells until the point to turn them into pro-tumorigenic cells. The tumor microenvironment can also affect the activity of non-immune cells, such as fibroblasts [77], influencing the natural course of tumors and the outcome of anti-cancer treatments.

## 3. Conclusions

In conclusion, with this mini-review, we attempt to shed a glimmer of light on the intricate network occurring between the protective responses put in place by cancer cells to resist constitutive stress and to adapt to that induced by anti-cancer treatments. In particular, we highlighted the interplay between UPR and DDR and how these responses cross-talk with other adaptive processes, which are also essential for cancer cell survival. Based on the evidence so far, it appears that targeting each response may have an impact on the others and that interrupting the interconnections between them and/or the molecular pathways regulating these responses might help to design more effective therapeutic strategies to be utilized in the tough battle against cancer.
